# The effect of complex cognitive context on the dynamic stability during gait initiation in older women

**DOI:** 10.3389/fnagi.2023.1342570

**Published:** 2024-01-11

**Authors:** Yuxia Chen, Hongyuan Tang, Yuanxin Wang, Chunxia Jin, Lihong Wang, Wensheng Miao, Xiangdong Wang

**Affiliations:** ^1^Key Laboratory of Exercise and Health Sciences of Ministry of Education, School of Exercise and Health, Shanghai University of Sport, Shanghai, China; ^2^Henan Sports Science and Technology Center (Henan Anti-Doping Center), Zhengzhou, Henan, China; ^3^Henan Provincial Third People's Hospital, Zhengzhou, Henan, China; ^4^Huanghe Science and Technology College, Zhengzhou, Henan, China; ^5^School of Physical Education, Zhengzhou University, Zhengzhou, Henan, China; ^6^China Research Center on Aging, Beijing, China; ^7^School of Physical Education, Jimei University, Xiamen, Fujian, China

**Keywords:** gait initiation, anticipatory postural adjustments, dynamic stability, visual reaction time, cognitive context

## Abstract

**Background:**

Changes in cognitive control are considered potential factors affecting voluntary motor movements during gait initiation (GI). Simulating environments with higher cognitive resource demands have an effect on the stability of GI task performance, which is of significant importance for assessing fall risk in the older adults and devising fall risk management measures in multiple environments. This study aims to reveal the influence of complex cognitive competitive environment with increased cognitive demands on the dynamic stability during GI in the older women.

**Methods:**

Twenty-three older females and twenty-three younger females performed walking tests under three conditions: voluntary initiation (SI), visual light reaction time task (LRT), and cognitive interference + visual light reaction time task (C + LRT). Eight cameras (Qualisys, Sweden, model: Oqus 600) and three force plates (Kistler, Switzerland, model: 9287C) are used to obtain kinematic and kinetic data. To recorde the trajectory of center of pressure (CoP) and the position of the foot placement, and compute the anterior–posterior (A-P) and medio-lateral (M-L) dynamic stability at the onset and end moments of the single-leg support by means of center of mass (CoM) and gait spatiotemporal parameters.

**Results:**

Older women responded to the effect of complex environments involving cognitive competition on body stability by prolonging the lateral displacement time of the CoP during the anticipatory postural adjustments (APAs) phase, reducing step length and velocity, and increasing step width and foot inclination angle.

**Conclusion:**

Complex initiation environments lead to competition for cognitive resources in the brain, resulting in decreased stability of GI motor control in older adults. The higher the complexity of the cognitive resource demands environment, the lower the stability of GI in older adults, and the greater the effect on their M-L stability at the onset of stepping.

## Introduction

With age, there is a gradual decline in sensory, neural, and motor functions, leading to a decreased ability for the body to manage these competing demands (Patel et al., 2009). This decline in physical and cognitive abilities can easily trigger falls, which are a common social issue among older adults. For older individuals, when the body fails to produce timely and appropriate movement responses to unexpected events that disrupt balance, resulting in insufficient postural stability control, falls can occur. Responding with adaptive movements to environmental demands are crucial in fall prevention. Motor control primarily explores how humans maintain stability during whole-body movement tasks and how they adapt to environmental constraints (Martin et al., 1996). Research on motor control encompasses action, perception, cognition, as well as the interactions between individuals, tasks, and the environment (Shumway-Cook and Woollacott, 1995). Hauert posits that ‘motor function is a cognitive function’ (Hauert, 1986), and changes in the environment can alter the cognitive load and allocation of cognitive resources, resulting in changes in motor performance (Muralidharan et al., 2017). Investigating how healthy older adults adapt their movements in response to cognitive competition environments is essential for assessing fall risk in older adults and developing multi-environmental strategies of fall risk management. Although previous research has shown that cognition can influence movement control (Lord and Levy, 1994; Gentilucci and Chieffi, 2004; Shumway-Cook et al., 2023), a quantitative evaluation of how cognitive load and allocation of cognitive resources specifically affect the stability of motor control in older adults remains lacking.

Gait initiation (GI) is the brief process of moving from static standing on both feet to striding and walking, which requires the body to cope with the change in stability from static standing to stepping through a series of anticipatory postural adjustments (APAs) (Winter, 1995). Effective motor planning contributes to enhanced motor performance. However, when the initiation environment changes, the body needs to adapt to the external environment, especially for older adults, as this adaptive adjustment plays a crucial role in fall prevention. Constraints in gait adaptation may lead to falls (Shumway-Cook et al., 2023). GI is a process of autonomously disrupting stable posture and entering walking motor control, which is related to cognitive control (Boripuntakul and Sungkarat, 2017). There is an interaction between cognition and motor control, which influences GI motor performance (Bayot et al., 2018). Some studies have pointed out that simple reaction time paradigms can cause changes in brain cortical mechanisms during GI (Yazawa et al., 1997). Rhythmic visual cues can affect the allocation of cognitive resources during GI and preparation (Zhou et al., 2023). There are also studies indicating that some cognitive functions are associated with GI performance, and poorer visual spatial ability can lead to prolonged step time in older adults during GI (Boripuntakul and Sungkarat, 2017). However, the quantification of the effect of cognitive context on stability has not been discussed.

This study utilizes Hof’s gait stability assessment method (Hof et al., 2005; Hof, 2008) to quantify the movement stability during GI tasks under different cognitive resource demand context. Due to previous research indicating that gender is a major risk factor for falls in older adults, with older women being more prone to falling (Stevens and Sogolow, 2005; Johansson et al., 2016; Moreland et al., 2020; Centers for Disease Control and Prevention, 2023; Ministry of Civil Affairs of the People’s Republic of China, 2023), only older and young female participants were included in this study. We compared the dynamic stability of GI in elderly and young women under three conditions: self-initiated (SI), visual light reaction time task initiation (LRT), and cognitive interference + visual light reaction time task initiation (C + LRT). The aim of this research is to determine age-related differences in dynamic stability during GI processes, as well as the effect of different cognitive competition environments on motor stability during GI in older adults. Based on these two objectives, we hypothesized that older individuals would exhibit lower dynamic stability margin at the onset of stepping, as well as reduced anterior–posterior (A-P) and medio-lateral (M-L) direction stability at the completion of the step, compared to young individuals. Additionally, we assume that the dynamic stability during GI decreases with higher cognitive resource competition demands: SI > LRT > C + LRT.

## Materials and methods

### Study sample

Sample size was estimated by the software of G*power, which should an effect size (interaction Cohen *d*) of 0.3, at least 40 subjects are required. We assumed a 30% attrition rate. For a 80% statistical power (α = 0.05) to compere the significant difference, recruitment of 52 participants was needed. Finally, dropout samples and invalid data were excluded, the study involved 46 participants, which satisfied the requirement for statistical significance.

### Participants

Based on research findings, older women may be more susceptible to falls compared to older men (Johansson et al., 2016; Moreland et al., 2020; Centers for Disease Control and Prevention, 2023; Ministry of Civil Affairs of the People’s Republic of China, 2023). To minimize gender disparities, this study focused exclusively on female participants. The inclusion criteria for the participants were as follows: female, right-leg dominance, good physical health, absence of back and pelvic system disorders, neuromuscular disorders or balance impairments, and normal cognitive function.

This study used the Timed Up and Go (TUG) Test to assess walking and balance control (Beauchet et al., 2011; Savva et al., 2013), excluding participants with a high risk of falling (TUG test result ≥13.5 s) (Barry et al., 2014).

Individuals with an MMSE (Mini-mental State Examination, MMSE) functional score of >24 (Da Silva et al., 2023), frailty, lower limb injuries or surgeries within the past 6 months, a history of falls in the previous 2 years, and fatigue or poor condition prior to the experiment were excluded from the study.

All participants were informed about the specific test procedures and signed written informed consent forms. The study was approved by the Academic Ethics Committee of the School of Physical Education, Zhengzhou University.

### Instrumentations

Kinematic data were collected using eight three-dimensional near-infrared high-speed cameras (Qualisys, Sweden, model: Oqus 600) with a sampling frequency of 100 Hz. Fifty-two infrared reflective marker points with a diameter of 14 mm were attached to the participants for GI kinematic information. The placement method of the markers follows the Helen Hayes model. Dynamic testing was conducted using three Kistler force plates (90 cm × 60 cm × 10 cm, Switzerland, model: 9287C), with a sampling frequency of 1,000 Hz. A self-made remote control start signal lamp was used.

### Experimental procedures

The participants were instructed to refrain from engaging in strenuous physical activities for 3 days prior to the experiment and to avoid consuming stimulating beverages, such as alcohol or coffee, which might affect the nervous system on the day before the experiment. Sufficient sleep was ensured. On the day of formal testing, the participants were required to familiarize themselves with the experimental procedures and practice them thoroughly. After the experimental preparations were completed, participants performed a 5-min warm-up activity before proceeding to the formal testing.

The starting action involved participants standing on a force plate with standardized footwear and clothing, with each foot placed on a force plate. They were instructed to stand naturally in a static position with their feet shoulder width apart. The signal indicator is located at the endpoint, which is opposite to the walking direction of the subject, 1.2 m above the horizontal plane and 8 meters away from the starting position of GI.

The GI walking test was performed under three experimental conditions designed in this study, and the order of the three experimental conditions was randomly generated using a random number generator program. The specific conditions were as follows:

Condition 1 – SI: The participants stood naturally on the force plate for 3–5 s and then initiated a step forward voluntarily. Trials started with the subject in quiet standing with their feet placed in their preferred natural stance. Following a verbal cue of ‘anytime,’ subjects waited a self-selected time interval (no less than 3 s) then initiated forward stepping at their self-chosen pace. Five valid trials were collected and reviewed online to ensure a steady-state baseline before the ‘anytime,’ instruction. Trials without a period of steady-state standing were discarded and repeated.

Condition 2 – LRT: The participants stood naturally on the force plate, while the experimenter controlled the signal light and the participants were instructed to initiate a step forward when the light was turned on.

Condition 3 – C + LRT: The participants stood naturally on the force plate, and the experimenter verbally announced a randomly generated three-digit number. The participants were instructed to continuously state the number after subtracting 3 each time until the light was turned on, at which point they were to initiate a step forward.

### Data analysis

Data analysis was performed using Visual3D software (C-Motion, USA, v6.01.36) with Visual 3D software for gait analysis. Smoothing and denoising of kinematic and kinetic signals, using a low-pass filter with a cut-off frequency of 6 Hz for kinematics and 20 Hz for kinetics.

### Phase processes of gait initiation

**Figure 1:**
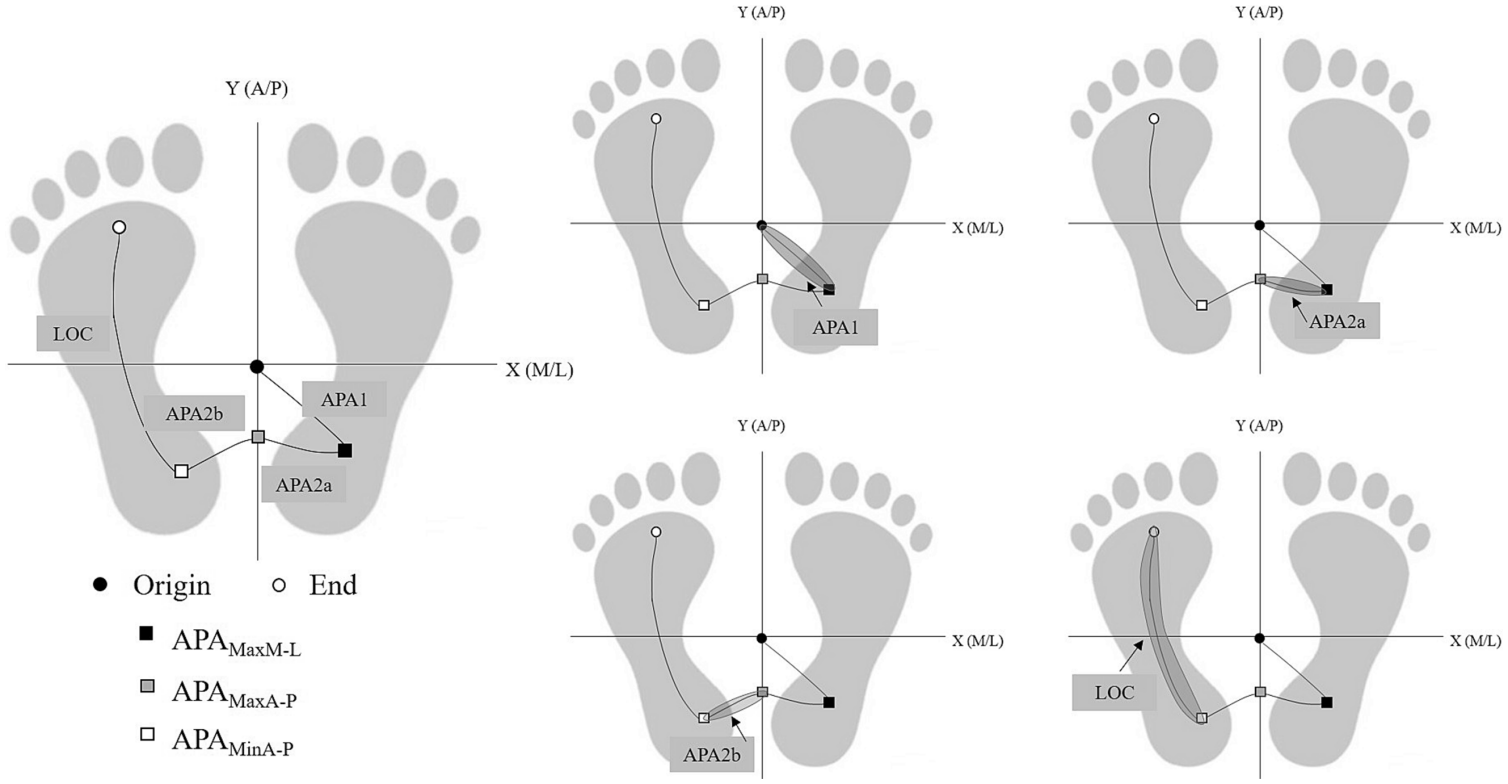
Phase division based on the keypoint-position of COP. Based on five key time points: (1) Origin; (2) APA_MaxM-L_; (3) APA_MaxA-P_; (4) APA_MinA-P_; (5) End, the phase of GI process is divided into four phases APA1, APA2a, APA2b, and LOC.

The GI process is divided into two phases: the APAs phase and the locomotion phase (LOC), refer to Figure 1 for details. The stability of the LOC process is analyzed in this study. The margin of stability (MoS) at the onset of LOC, as well as the forward and lateral control stability at the end of LOC are computed.

### Foot placement at the end of stepping

**Figure 2:**
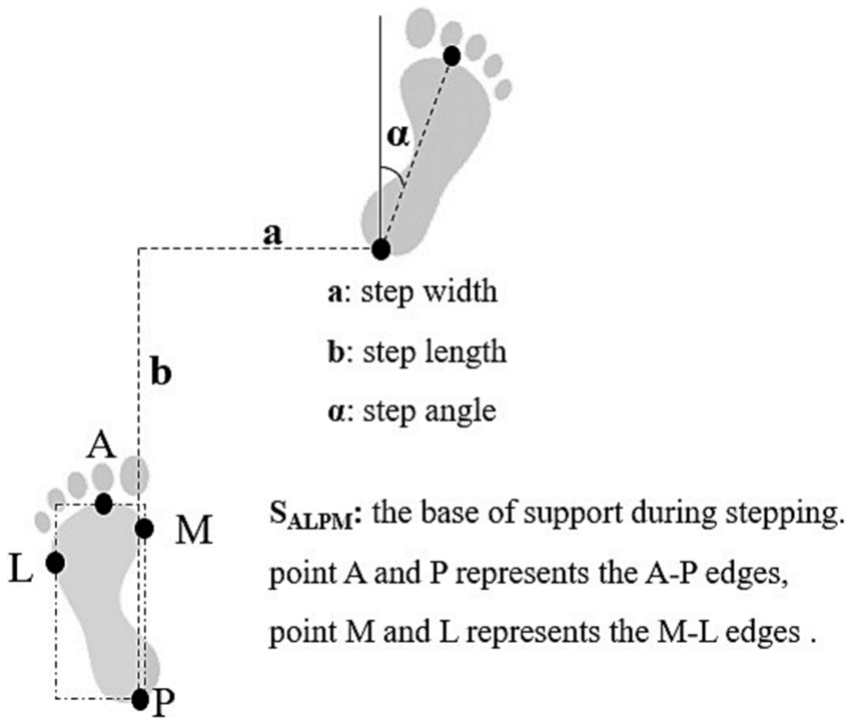
Base of support and position of foot placement.

We localized the foot placement at the end of stepping by the step length, step width, and foot declination angle, as detailed in Figure 2.

### Dynamic stability of gait initiation

#### Onset stepping of the locomotion phase

##### Margin of dynamic

Next to position CoM and velocity $V_{CoM}$ of the whole body CoM the ‘extrapolated center of mass’ (XcoM) can be introduced, see the Equation (1, 2) where omega0 is a constant related to stature. Based on the inverted pendulum model of balance, the XcoM enables to formulate the requirements for stable walking in a relatively simple form.


(1) $$\omega_{0}=\sqrt{g/l}$$

(2) $$\mathrm{XcoM}=CoM+\frac{V_{CoM}}{\omega_{0}}$$

Where $\omega_{0}$ is the M-L extrapolated CoM position accounting for CoM velocity, normalized by the eigenfrequency, where l is leg length measured from the lateral malleolus to greater trochanter, g is the gravity acceleration (Hof et al., 2005).

We computed the minimum $MOS_{\mathrm{ML}}$ and $MOS_{\mathrm{MAP}}$ are defined by Equation (3, 4)


(3) $$MOS_{\mathrm{ML}}=BoS\left(x\right)-\mathrm{XcoM}\left(x\right)$$

(4) $$MOS_{\mathrm{MAP}}=BoS\left(y\right)-\mathrm{XcoM}\left(y\right)$$

The $MOS_{\mathrm{ML}}$ and $MOS_{AP}$ were calculated as the distance between XcoM and the boundaries of the base of support (BoS) during single stance from the foot markers, the fifth metatarsal-phalangeal joint for the lateral border and the medial malleolus for the medial border (Hof et al., 2005; Osada et al., 2022). See Figure 2 for illustration of support surface edges.

#### End of the locomotion phase

##### Lateral stability control

We calculated the lateral stability with a constant offset control by positioning the right foot $B_{\mathrm{ML}}$ to the right of the XcoM, in which is related to the desired step width W (Hof, 2008) as Equation (5).


(5) $$B_{\mathrm{ML}}=\frac{W}{e^{\omega_{0}t}+1}$$

Where $B_{\mathrm{ML}}$ is the stability of lateral control, W is step width, t is the time of LOC.

##### Forward stability control

The simplest stable control of CoP position could be made by positioning the CoP at a constant distance behind the XcoM ‘constant offset control,’ in which the offset $B_{AP}$ was a constant distance, calculated from Equation (6) (Hof, 2008) as.


(6) $$B_{AP}=\frac{L}{e^{\omega_{0}t}-1}$$

Where $B_{AP}$ is the stability of forward control, L is step length, t is the time of LOC.

### Statistical analysis

Statistical analysis was performed using SPSS (version 22, SPSS Inc., Chicago, IL USA). Shapiro–Wilk test was used to verify if the parameters were normally distributed; the parameters were not normally distributed, so we used Mann–Whitney *U* test for comparing the data of the older and young groups. Between group and three context were used in Multivariate Analysis of Variance (MANCOVA) to compare group differences. Effects were considered to be significant at *p* < 0.05.

## Results

### Sample characteristics

**Figure 3:**
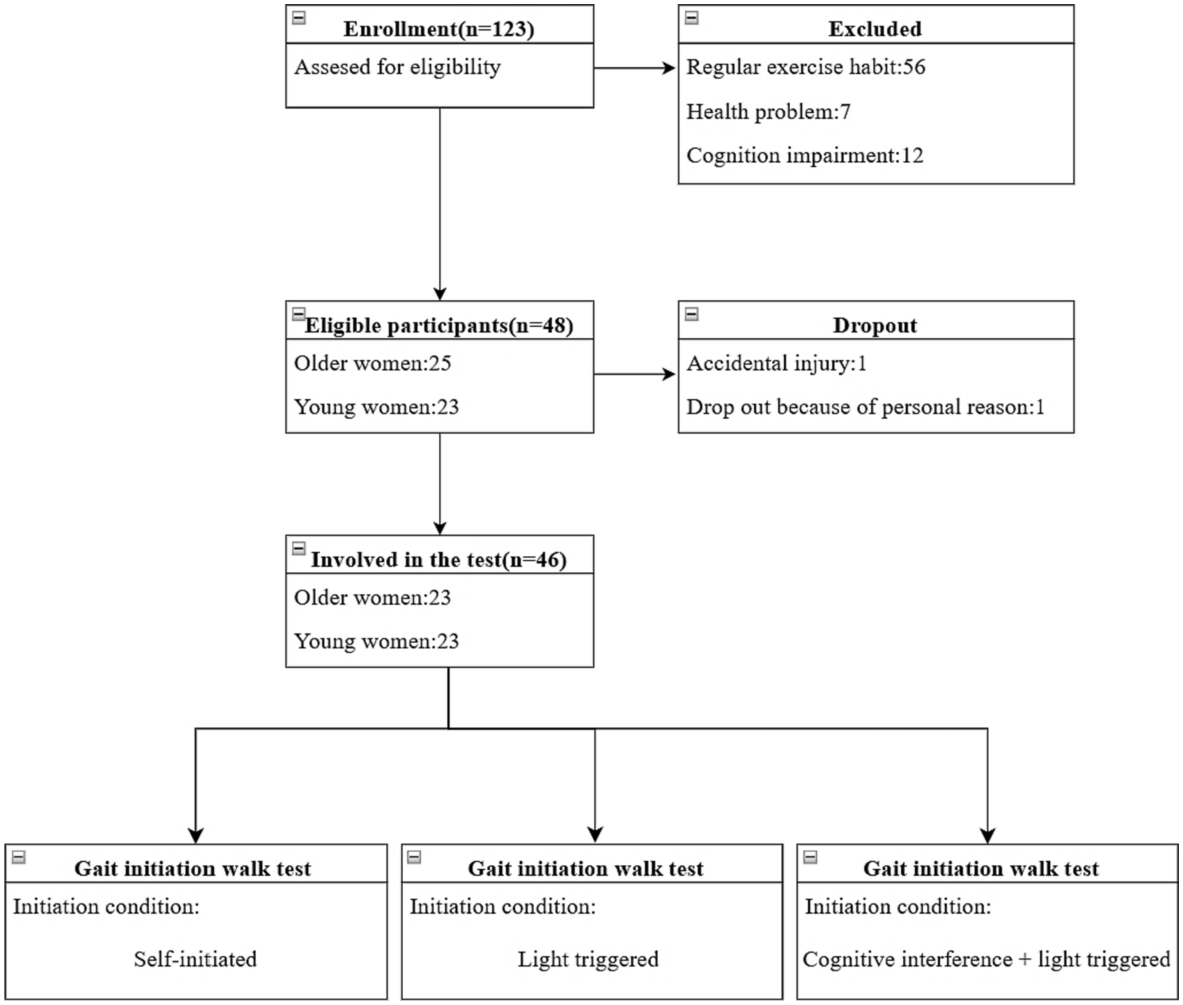
Recruitment of participants in older women and young women.

In total, the study included 23 older females who did not engage in regular exercise and 23 young females were included in the study. The recruitment process for participants of both surveys is illustrated in Figure 3. The demographic information of the study participants and their performance on the MMSE and TUG tests are presented in Table 1.

**Table 1:** Information of participants.

	Older Mean ± SD	Young Mean ± SD	*p*-value
*n* (female) Age(years)	23 65.59 ± 3.66	23 20.86 ± 0.96	**<0.001**
Height(m)	1.61 ± 0.53	1.63 ± 0.32	0.404
Mass(kg)	65.41 ± 12.25	62.35 ± 7.01	0.072
BMI	23.33 ± 2.46	22.08 ± 2.93	0.055
MMSE scores	28.0 ± 0.40	28.8 ± 0.44	0.286
TUG(s)	8.98 ± 0.98	8.84 ± 0.54	0.482

Significant effects are marked in bold.

### Duration of each phase during gait initiation

**Figure 4:**
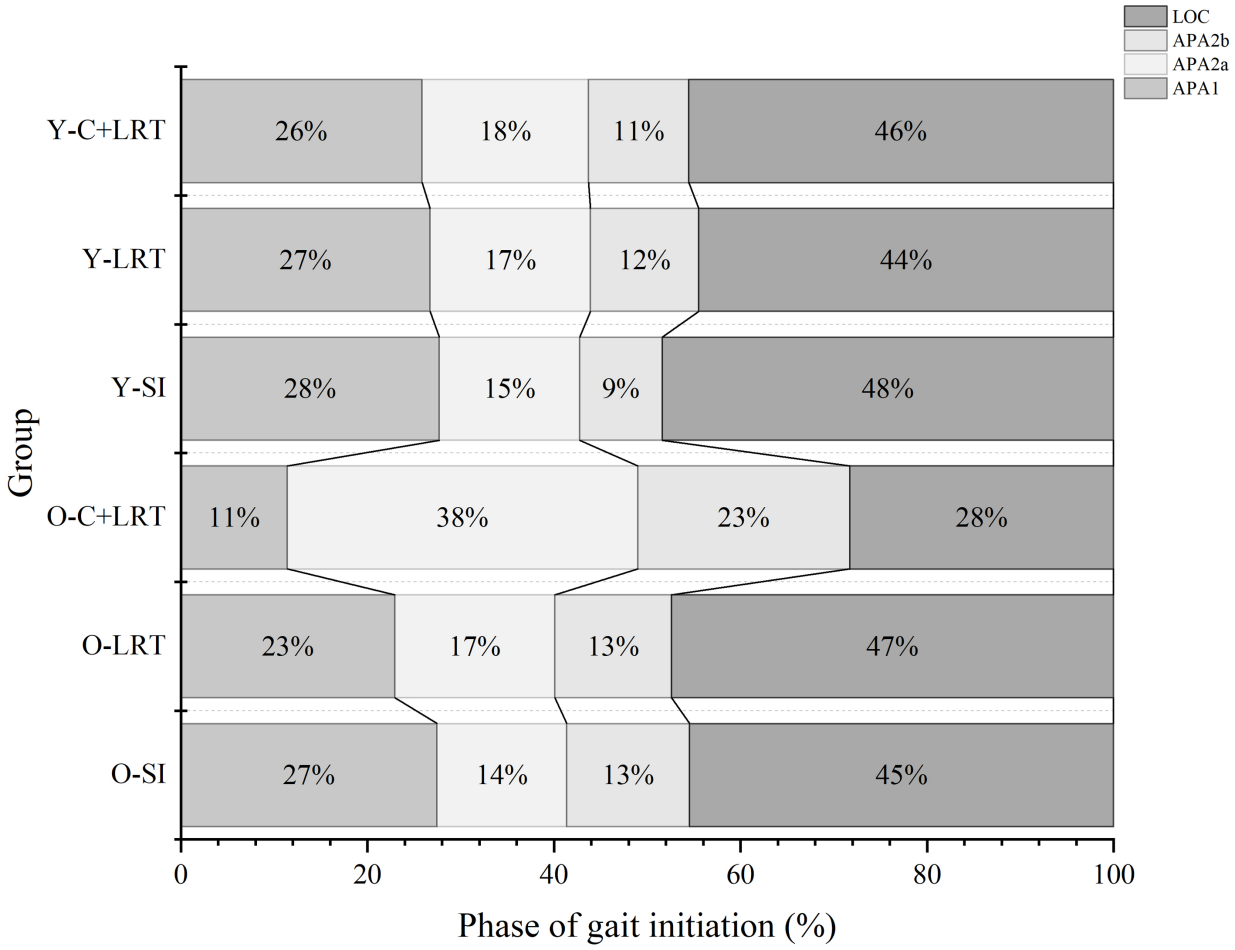
Duration of each phase. Figure illustrates the normalization of the entire GI process: APA1, APA2a, APA2b, and LOC. The percentages of time process for each phase are displayed from left to right in every line. O-SI, older self-generated; O-LRT, older light reaction time; O-C + LRT, older cognitive interference + light reaction time; Y-SI, young self-generated; Y-LRT, young light reaction time; Y-C + LRT, young cognitive interference + light reaction time.

By normalizing the entire GI process, we obtained a diagram illustrating the time ratios of each phase. As shown in the Figure 3. In all three conditions, the duration sequence in the young group is LOC > APA1 > APA2a > APA2b, which is consistent. However, in the older group, this pattern is only observed in the SI and LCT conditions, whereas under C + LCT conditions, the durations of the different phases undergo changes, with shorter durations for APA1 and LOC, and longer durations for APA2a and APA2b. There are statistically significant in the duration of the APA1 phase between SI and C + LCT in both older women (*p* < 0.001, 95%CI: 46.69, 72.85) and young women (*p* = 0.001, 95%CI: 10.24, 37.55). Additionally, there are significant differences in the duration of the LOC phase between SI and C + LCT in older adults (*p* = 0.028, 95%CI: 0.40, 6.69) (Figure 4).

### Dynamic stability

**Figure 5:**
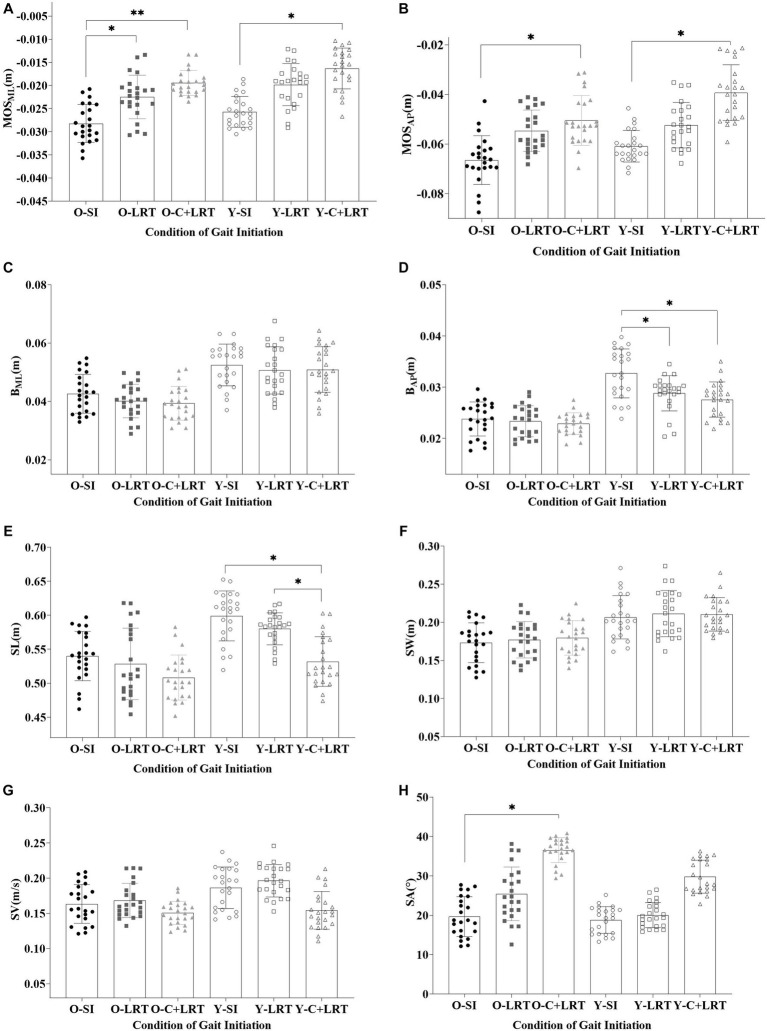
Dynamic stability and foot placement. (O-SI, older self-generated; O-LRT, older light reaction time; O-C + LRT, older cognitive interference + light reaction time; Y-SI, young self-generated; Y-LRT, young light reaction time; Y-C + LRT, young cognitive interference + light reaction time).

The results presented in Table 2 indicate a consistent trend in the M-L and A-P stability at the start and end of gait, with SI < LRT < C + LRT, older adults exhibit lower stability compared to young adults. Statistical analysis indicates significant differences in both M-L and A-P stability among older and young adults at the beginning of stepping under the SI and C + LRT conditions. For older adults, statistically significant differences are observed in M-L stability under the SI and LRT conditions, as well as between SI and C + LRT conditions. Young adults also demonstrate statistically significant differences in A-P stability at the end of stepping under the SI and LRT, as well as SI and C + LRT conditions. The three conditions significantly effect on the M-L and A-P stability at the start of gait, while no significant effects are observed between different age groups. At the end of gait, both older and young adults display significant effects on M-L and A-P stability, with all three conditions significantly affecting A-P control stability. There is no significant interaction between age group and walking condition concerning observed stability values. For detailed statistical results, please refer to Figure 5.

**Table 2:** Dynamic stability on the onset and end phase of locomotion.

	SI		LRT		C + LRT		*P-value*		
	Older	Young	Older	Young	Older	Young	Group	Condition	Group * condition
	M ± SD	M ± SD	M ± SD	M ± SD	M ± SD	M ± SD			
MoS_ML_(m)	−0.028 ± 0.007	−0.026 ± 0.005	−0.022 ± 0.009	−0.02 ± 0.01	−0.019 ± 0.008	−0.016 ± 0.007	0.130	**<0.001**	0.989
MoS_AP_(m)	−0.067 ± 0.035	−0.061 ± 0.022	−0.055 ± 0.024	−0.052 ± 0.014	−0.05 ± 0.026	−0.039 ± 0.016	0.271	**0.020**	0.821
B_ML_(m)	0.043 ± 0.006	0.053 ± 0.007	0.04 ± 0.007	0.051 ± 0.01	0.039 ± 0.006	0.05 ± 0.01	**<0.001**	0.144	0.841
B_AP_(m)	0.024 ± 0.002	0.033 ± 0.009	0.023 ± 0.003	0.029 ± 0.003	0.022 ± 0.002	0.028 ± 0.003	**<0.001**	**0.002**	0.453

SI, LRT, and C + LRT represent the three walking conditions, respectively. MoS_ML_, MoS_AP_ represent the M-L and A-P directional stability when right toe-off after APA completion, respectively. B_ML_ and B_AP_ indicate the M-L and A-P directional stability when right heel strike at the end moment of LOC, respectively. Significant effects are marked in bold.

### Foot placement

As shown in Table 3, we found that the step length and width of older individuals were smaller than young individuals’ under all three walking conditions, with step length- SI > LRT > C + LRT and step width- SI < LRT < C + LRT. The walking speed of the older was also slower than that of the young, with C + LRT > SI > LRT. Additionally, the foot inclination angle of the older was greater than that of the young, with SI < LRT < C + LRT. There were statistically significant differences in step length between young individuals during the SI and C + LRT conditions, as well as between LRT and C + LRT conditions. Similarly, there were statistically significant differences in foot adduction angle between older individuals during the SI and C + LRT conditions. For more detailed information, please refer to Table 3.

**Table 3:** Foot placement at the termination of gait initiation.

	SI		LRT		C + LRT		*P-value*		
	Older	Young	Older	Young	Older	Young	Group	Condition	Group * condition
	M ± SD	M ± SD	M ± SD	M ± SD	M ± SD	M ± SD			
SL(m)	0.539 ± 0.053	0.599 ± 0.047	0.526 ± 0.042	0.582 ± 0.064	0.507 ± 0.052	0.532 ± 0.017	**<0.001**	**0.003**	0.403
SW(m)	0.173 ± 0.031	0.205 ± 0.032	0.178 ± 0.035	0.213 ± 0.031	0.18 ± 0.025	0.211 ± 0.036	**<0.001**	0.655	0.999
SV(m/s)	0.163 ± 0.028	0.186 ± 0.029	0.168 ± 0.024	0.196 ± 0.023	0.151 ± 0.017	0.154 ± 0.027	0.654	0.334	0.708
SA(°)	19.67 ± 13.342	18.791 ± 11.572	25.433 ± 16.738	20.028 ± 15.698	36.548 ± 13.156	29.794 ± 12.099	0.327	**0.030**	0.840

SI, LRT, and C + LRT represent the three walking conditions, respectively. SL, step length; SW, step width; SV, velocity of stepping; SA, step angle. Significant effects are marked in bold.

## Discussion

In the context of studying postural instability and fall risk, GI is utilized frequently as an objective measure. It is observed that falls are more likely to occur during transitional movements. Consequently, assessing GI serves as a method to gauge postural instability and fall risk. In order to address compromised balance, individuals naturally and effectively take a step. The central nervous system (CNS) employs stable and efficient mechanisms to cope with the inherent instability associated with GI. The CNS is capable of adapting and predicting limitations within the motor system and environment while performing GI actions. By continuously comparing expected and actual movements in the environment, the CNS modifies movement control methods to identify the most effective and efficient approach to achieve the goal (Hadders-Algra, 2010). Nikolai Bernstein highlighted that there is no fixed solution to a motor problem, but rather a movement pattern that emerges as a consequence of constantly changing constraints (Chernavsky and Talis, 2021). Coordinated movement control in the human body occurs through the synthesis of individual, environmental, and task-specific constraints. Autonomous GI involves internal self-perturbations in balance, resulting in adaptations of the BoS and the transition from static standing to dynamic walking. The Choice Stepping Reaction Time test, which assesses an individual’s capacity to promptly initiate and execute a stepping task, has proven to be a reliable predictor of future fall risk (Yiou et al., 2016). This study aimed to investigate the impact of cognitive resource competition on GI stability in older adults by increasing the complexity of the reaction time environment. The purpose was to observe the adaptive movement adjustments made by older adults in response to environmental constraints.

### Duration of each phase during gait initiation

The M-L direction movement during GI reflects the body’s stable control, while the A-P direction movement can predict motor performance. Our study results (Figure 3) revealed that the C + LRT walking condition, which involves higher cognitive resource competition, significantly effected older adults by increasing the time of CoP displacement in the M-L direction (APA2a and APA2b). As the complexity of the walking initiation conditions increases, attention is diverted by other brain functions during the expected postural adjustment stage of GI (APA1, APA2a, and APA2b processes). This diversion causes a shorter displacement time of CoP towards the right rear to generate propulsion force and longer durations for APA2a and APA2b processes to ensure more time is allocated for body lateral transfer and increased stability. In the subsequent step process, older adults reduce the step duration (LOC process) to compensate for the time required for lateral body weight shift. The influence of the environment on GI is reflected in these anticipated and executed stages: faster gait requires longer anticipation time and shorter execution time (Brenière et al., 1987). Previous studies have interpreted increased LOC duration as an improvement in adaptability to stability function (Cau et al., 2014). However, under C + LRT conditions, older adults significantly reduce LOC duration, potentially compromising their movement stability and increasing fall risk. This highlights the need for future research on how older adults respond to the loss of stability through rapid and coordinated postural adjustments under cognitive interference states. The human body reacts to disturbance-induced stability loss by swiftly and harmoniously adjusting posture, which can be categorized as either ‘APAs’ or ‘compensatory postural adjustments (CPAs)’ (Santos et al., 2010). The CNS employs posture adjustment strategies to neutralize balance disturbances caused by APAs and CPAs. Their purpose is to maintain posture in the event of disturbance or imbalance, facilitating balance control and safe performance of daily activities (Gélat et al., 2018; Delafontaine et al., 2019). The CNS anticipates potential consequences of upcoming movements, and these motor predictions (APAs) counteract any potential destabilizing effects caused by the movement itself or external perturbations, ensuring effective postural control and stability (Miall and Wolpert, 1996). Previous studies have demonstrated the CNS is capable of adjusting posture based on its internal resources to adapt to external environmental conditions (Yiou et al., 2016). Our study extends these findings by providing additional evidence that the environment can influence GI control. Specifically, in environments with complex cognitive resource competition, older adults employ a compensatory postural adjustment strategy to ensure stable body control. This strategy involves reducing the time needed for CoP displacement towards the right rear for generating propulsion force, prolonging body lateral displacement duration, and diminishing step duration during stepping.

### Dynamic stability

The process of GI requires two skills: forward propulsion and balance control, accompanied by posture-locomotion coupling, where posture events take precedence over movement execution (Mille et al., 2014). Two biomechanical requirements for successful GI are the generation of momentum (in the forward and supporting leg directions) and the maintenance of balance (Polcyn et al., 1998). The lifting and stepping forward of the swing leg can potentially cause lateral imbalance in the body, and the CNS employs stable and efficient mechanisms to deal with inherent instability during GI. This is achieved partly through the CoP movement towards the swinging leg via APA, which moves the CoM towards the supporting leg, and partly compensating through effective stepping. The most commonly used metric for quantifying dynamic stability during gait is the ‘MoS’ proposed by Hof et al. (2005). MoS is a composite variable that considers the relationship between the CoM position, velocity, and the BoS, and has been widely adopted for quantifying stability during GI tasks. It is a measure of walking stability derived from dynamic stability theory and the human inverted pendulum model, representing the shortest distance from a given CoM position-velocity state point to the posterior imbalance boundary. Gait stability is evaluated by quantifying the MoS in the A-P and M-L directions, with the M-L MoS value considered a key indicator of walking stability. The complexity of the environment can affect the stability of GI, leading to decreased stability at the beginning and end of the stepping phase. Older adults exhibit lower stability than young adults in complex environments. At the initiation of stepping, older adults ensure stability during the transition from double-leg to single-leg support through longer durations of APAs, with lower stability observed in the M-L direction compared to the A-P direction. Additionally, complex environments result in a substantial decrease in the M-L stability control among older adults. Nakano et al. (2016) suggested that age-related changes in lateral balance control under time constraints may increase the risk of falls during GI. Time constraints can alter the postural stability in the A-P and M-L directions, with the human body adjusting step length and foot placement during swing to ensure safe and efficient stepping. Time-pressured GI test is commonly used for assessing fall risk in frail individuals, those with neurological and musculoskeletal disorders. Reaction time tests can be used to evaluate an individual’s ability to quickly trigger and execute stepping tasks, with reaction time being considered a predictor of future fall risk (Yiou et al., 2016). Older adults may adjust their stability control at the end of the stepping phase by adjusting their foot placement. Without time constraints, individuals prioritize forward propulsion, followed by stability, and then energy consumption (Moraes et al., 2004).

It is challenging to study action execution, working memory, and attention independently due to their intimate relationship with cognitive processes. Action execution function involves advanced cognitive processes involved in non-routine, goal-directed behavior, including action initiation, response inhibition, planning, processing multiple information sources, and response monitoring (Bayot et al., 2018). Pellecchia (2003) suggests that the swing of the CoP during the APAs phase increase with attentional demand during cognitive tasks, even during stance control, indicating that even the dynamic adjustments of postural control during standing require a certain level of attentional resources. When attentional resources are depleted by a stimulus task, posture control is impaired, resulting in inefficient posture adjustments that lead to ‘over or under-corrections’ and increased sway. When attentional resources are depleted by other tasks, it causes increased variability in stride length among older adults (Nakano et al., 2016). Sun and Shea demonstrated that GI performance during dual-task walking is influenced not only by task instructions and environmental factors but also by task priority, which is related to the complexity of concurrent cognitive tasks (Sun and Shea, 2016). These studies suggest that GI training during dual-task can be used as a fall prevention strategy for older adults. Clinical gait assessments should include GI and cognitive evaluations, with rehabilitative strategies aimed at improving cognitive function and GI ability, potentially reducing fall risk.

### Foot placement

The ability to adapt to variable and unpredictable environmental changes during gait requires continuous modification of walking parameters, reorientation of posture direction, and stability recalibration. Coarse control of balance during the GI process is achieved through the placement of the swing leg foot (Jian et al., 1993), which is actively adjusted by the CNS to maintain body’s lateral balance (Bauby and Kuo, 2000; Donelan et al., 2004). Humans maintain gait stability by controlling the placement of the feet on the M-L side in coordination with the CoM. The CNS anticipates the initial lateral movement of the body’s orientation and magnitude to ensure that it corresponds to the expected position of the M-L foot placement. By altering the posture before stepping forward, the foot position is adapted to ensure stability upon landing (Mille et al., 2007). Even if there is a slight misplacement of the foot at the time of landing, it can still be compensated for by adjusting the M-L position of the CoP, a mechanism known as the ‘ankle strategy’ (Hof et al., 2007). Reimann et al. (2017) proposed that foot placement and ankle strategy may be two independent mechanisms that are coupled and coordinated temporally to complement the M-L instability of APAs and the stiffness of the stance leg, thereby maintaining frontal plane stability of the body (Van Dieёn et al., 2008). Furthermore, studies have shown that visual gaze behavior influences the foot placement strategy during walking (Bonnen et al., 2021).

Our findings indicate that older adults control their A-P and M-L stability during GI and end by shortening their step length, increasing their step width, decreasing their step speed, and increasing their foot progression angle. This aligns with previous studies showing that with age, older adults exhibit significantly reduced step length and speed during GI (Muir et al., 2014). Some studies have found that older fallers demonstrate a significantly shorter first step length during the GI process (Azizah Mbourou et al., 2003). Under time constraints, age affects dynamic balance control during stepping adjustments by either increasing or shortening step length (Nakano et al., 2016). Disruptions in frontal plane posture control prompt motor adjustments to maintain stability and efficiency during the first step (e.g., maintaining step length). The time sequence from the onset of the M-L APA to the start of stepping may indicate a feedback adaptation reflecting the conditioned posture state used to trigger stepping action; stepping action initiated with M-L APA assistance starts faster (Mille et al., 2007). Furthermore, it has been suggested that fear of falling reduces available attentional resources (Uemura et al., 2012). Older adults exhibit shorter step length, stride length, and slower step velocity after disturbances, with highly fearful older adults demonstrating greater changes (Bueno et al., 2019). The CNS primarily employs muscle synergy activation to send central commands predicting postural adjustments, and when facing different postural threats, the human body modifies APAs strategies by controlling the active-antagonist muscle activation of different lower extremity joints; the co-activation of CPAs muscles decreases to ensure greater redundancy for executing CPAs and re-establishing postural stability as needed (Cesari et al., 2022). When faced with postural threats, fear of falling increases conscious control over balance, and the CNS adjusts APAs and CPAs to minimize the risk of falling. Fear of falling causes older adults to exhibit cautious gait patterns, and APAs during GI can be used to quantify the severity of fear of falling. Studies examining the influence of gait speed on the control of M-L dynamic stability control on the M-L side during gait suggest that increased risk of falls due to increased lateral displacement can be compensated by a wider step width (Caderby et al., 2014). Considering the importance of appropriate foot placement during GI and gait recovery responses, variations in the length of the first step in older adults may be a significant predictor of postural issues.

### Limitations

This study focused on quantifying the stability control during the single-leg stance phase of GI, without examining the coordination of limb spatial positions or adjustments to ground reaction forces. It considered the influence of cognitive resource competition on GI stability, but task prioritization was not taken into account. Additionally, due to previous research indicating that gender is a major risk factor for falls in older adults, with older women being more prone to falling (Qu, 2015; Johansson et al., 2016), only older and young female participants were included in the study, with no male participants, and no age-grouping of elderly females was performed.

## Conclusion

GI serves as a typical functional task for studying balance control mechanisms and stability characteristics in complex whole-body movements. The complexity of the initiation environment leads to competition for cognitive resources in the brain, affecting the stability of GI in older adults. As environmental complexity increases, GI stability decreases in older adults, with a greater effect from the complexity of the environment on the M-L stability at the onset of stepping. Older adults adapt to the effect of cognitive competition in complex environments on their body stability by prolonging the M-L displacement of the CoP during the APAs phase, reducing step length and walking speed, and increasing step width and foot progression angle. Due to age-related physiological declines in neural, muscular, and skeletal systems, older adults’ dynamic balance posture control ability decreases. The processing capacity of the brain is limited, and when individuals are required to process too much information, too many tasks, or too many goals, processing speed or accuracy decreases. For research on dynamic balance control in older adults, selecting the GI, a natural movement pattern that is prone to falls, and studying the action initiation strategies in environments with cognitive resource competition, can further enhance the understanding of fall prevention strategies in older adults, providing objective references for fall risk management in various environments.

## Data availability statement

The original contributions presented in the study are included in the article/supplementary material, further inquiries can be directed to the corresponding authors.

## Ethics statement

The studies involving humans were approved by Academic Ethnic Committee of the School of Physical Education, Zhengzhou University. The studies were conducted in accordance with the local legislation and institutional requirements. The participants provided their written informed consent to participate in this study. Written informed consent was obtained from the individual(s) for the publication of any potentially identifiable images or data included in this article.

## Author contributions

YC: Writing – original draft, Writing – review & editing. HT: Investigation, Resources, Writing – review & editing. YW: Resources, Supervision, Writing – review & editing. CJ: Resources, Writing – review & editing. LW: Resources, Writing – review & editing, Data curation. WM: Writing – review & editing, Resources. XW: Funding acquisition, Writing – review & editing.
